# Correction: A Novel Copper Chelate Modulates Tumor Associated Macrophages to Promote Anti-Tumor Response of T Cells

**DOI:** 10.1371/journal.pone.0117629

**Published:** 2015-01-28

**Authors:** The PLOS ONE Staff

The eighth author’s name is spelled incorrectly. The correct name is: Sudipto Ganguly
